# The Possible Suspension of Old Age

**Published:** 1885-11

**Authors:** 


### The Possible Suspension of Old Age.

Dr. S. W. Caldwell thus writes in the *Mississippi Valley Medical Monthly:*

In bygone times those profound mysteries and metaphysicians, the Rosicrucians, and still later the Alchemists, claimed to have discovered the elixir of life, They asserted that the old age might be retarded and life considerably prolonged by means of an elixir, preventing or rather suspending physical decay. The celebrated Rosicrucian, Dr. Hood, whose writings became famous, is said to have reached a hundred years.

Modern science has recently made more startling discoveries than were those dreamed of by the alchemist. The possibility of prolonging life has throughout all ages been deemed worthy of notice by great thinkers, among whose number the illustrious Bacon and Hufland are enrolled. In the following remarks I shall endeavor to give the latest scientific knowledge relative to this interesting and, in some respects, novel subject.

Premature age, as engendered by various mental and physical causes, excesses, etc., does not come within the scope of this short paper. The principal characteristics of old age, as demonstrated by anatomical research, are a deposition of fibrinous, gelatinous, and earthy material in the system. Every organ of the body, during old age, is especially prone to ossific deposits. The earthy deposits have been found to consist primarily of phosphates and cararbonates of limes combined with other calcareous salts.

According to the researches of Dr. Williams, of England, man begins in a gelatinous and ends in an osseous or bony conditions. From the cradle to the grave a gradual process of ossification is undoubtedly present; but after passing middle age the ossific tendency becomes more markedly developed until it finally ushers in senile decrepitude. These earthy deposits during old age materially interfere with the due performance of function by the organs; hence we find imperfect circulation in the aged; the heart gradually becomes ossified, the large blood vessels blocked up with calcareous matter, and nutrition hindered.

A distinguished physiologist says: "If repair was always equal to waste, life would only terminate by accident" And it is the opinion of eminent scientists that the majority of all who pass sixty-five years suffer more or less from these ossific deposits. Therefore, bearing these facts in mind, we plainly see that the real change which produces old age is in truth nothing more or less than a slow but steady accumulation of calcareous matter throughout the system; and it is owing to these deposits that the structure of every organ is altered, tnus elasticity giving way to senile rigidity. Blockage of various organs thus commenced, and sooner or later a vital part becomes involved, and death of necessity follows. The idea that old age was brought about simply, or at all, by a decline of the vital principle has long since been discarded by scientists, and the true cause found to be that of gradual disintegration of the tissues because of the inadequate supply of blood.

Again, quoting from Dr. Williams, this process is believed to be of a chemical nature, consisting in the concretion and accumulation of calcareous salts, phosphates and carbonates of lime. The causes of old age therefore being nothing more nor less than ossific deposits, let us for a moment look for the causes and influences leading to the condition we have described; for having arrived at the predisposing causes of senile decay, it yet remains for us to go still further, and seek out their origin.

The two principle causes of old age are, first, fibrinous and gelatinous substances; and, second, calcareous deposits. According to recent researches of Delany Evins, the origin of the first, the fibrinous and gelatinous, may undoubtedly be traced to the destruction of atmospheric oxygen, and demonstrable by the following argument:

In the air we breathe the relative proportion of oxygen to nitrogen is 22 to 78; and although oxygen is in far smaller bulk, yet it is the most active element. Now, oxygen has an affinity for every other element except fluorine; and as oxygen plays by far the most important part in these chemical changes constantly at work in the economy, life itself is but a constant waste by oxidation and reparation by food. In the blood exists albumen and fibrin, themselves resolved into component parts, carbon, hydrogen, nitrogen, oxygen, sulphur and phosphorus. Fibrin, it is claimed, contains one and five-tenths more oxygen than albumen. Now, oxygen converts albumen into fibrin, fibrin itself being but an oxide of albumen. Although unquestionably fibrin nourishes the organs of our body, yet it becomes at times, as we reach the cool and shady walks in the evening of life, accumulated in redundant quantity, blockading the streams of life as doth the chilling winds of winter the mountain rivulets. There is always a struggle going on in our bodies between accumulation and elimination, and thus it is that the fibrinous and gelatinous accumulations of old age are chiefly traceable to chemical action of atmospheric oxygen.

The calcareous deposits next claim our attention, being proved by anatomical investigation to be peculiarly characteristic of old age. In the human body water forms seventy per cent. of its aggregate weight; in fact, there is not a single tissue but contains water as a necessary ingredient. Now, water holds certain salts in solution, which become more or less deposited, notwithstanding the large proportion eliminated through the secretions. Nevertheless, it is but a question of time before these minute particles deposited by the blood have a marked effect in causing the stiffness and aridity of advanced life. The reason why in early life the deposit of earthy matter or salts is so infinitesimal is simply because they have not had time to accumulate. Besides providing the requisite elements of nutrition, food contains calcareous salts, which upon being deposited in the arteries, veins and capillaries, become the proximate cause of ossification and old age. Mr. Lewis, an eminent English scientist, in his Physiology of Common Life, says: "Moreover, in food we are constantly introducing different substances which produce variation in the nutrition of the parts. These different accumulations exert their influence in those changes named age, and they culminate in the final change named death."

Having now traced the primary existence of calcareous matter to food itself, it is consequently a subject of no small moment to ascertain the various dietetic articles containing these salts. As a matter of fact, everything we eat does contain them to a greater or less degree. The cereals are found most rich in them; so bread itself, the so-called staff of life, except in great moderation, most assuredly favors the deposition of these salts in the system. The more nitrogenous our food, the greater its percentage of calcareous matter; thus a diet composed principally of fruit, from its lack of nitrogen, is best adapted for preventing or suspending ossification.

Moderation in eating must ever be of great value as an agent for retarding the advent of senile decay. Large eaters more rapidly bring on ossific deposits by taking in more than is utilized or excreted, naturally resulting in blockading the vessels and destroying their normal functions. According to the best authority I have been able to consult, the following seem to be the best articles of food, as containing the least of earthy salts: Fruit, fish, poultry, flesh of young mutton and beef, because, as before stated, of their being less nitrogenous. Fluids, as part of the diet, are of special import. All well and spring water contains considerable of the earthy salts, and should therefore be avoided, and cistern water used in its stead, because water is the most universal solvent known. Therefore, if when taken into the system clear of foreign matter, it is to that extent the better prepared to dissolve and take up those earthy salts, and convey them out of the system. The addition of fifteen or twenty drops of dilute phosphoric acid to the glass of water, and drank three times a day, will add to the solubility of these earthy salts.

